# Mobile health, exercise and metabolic risk: a randomized controlled trial

**DOI:** 10.1186/1471-2458-14-1082

**Published:** 2014-10-18

**Authors:** Robert J Petrella, Melanie I Stuckey, Sheree Shapiro, Dawn P Gill

**Affiliations:** Department of Family Medicine, Centre for Studies in Family Medicine, Western Centre for Public Health and Family Medicine, Western University, (2nd Floor), 1151 Richmond St., London, Ontario N6A 3K7 Canada; Lawson Health Research Institute, Aging Rehabilitation and Geriatric Care Research Centre, London, Ontario Canada; Faculty of Health Sciences, Western University, London, Ontario Canada; School of Public Health, University of Washington, Seattle, WA USA

**Keywords:** Mobile health, Metabolic syndrome, Exercise prescription, Exercise intervention, Disease prevention, Rural health

## Abstract

**Background:**

It was hypothesized that a mobile health (mHealth) intervention would elicit greater improvements in systolic blood pressure and other cardiometabolic risk factors at 12 weeks, which would be better maintained over 52 weeks, compared to the active control intervention.

**Methods:**

Eligible participants (≥2 metabolic syndrome risk factors) were randomized to the mHealth intervention (n = 75) or the active control group (n = 74). Blood pressure and other cardiometabolic risk factors were measured at baseline and at 12, 24 and 52 weeks. Both groups received an individualized exercise prescription and the intervention group additionally received a technology kit for home monitoring of biometrics and physical activity.

**Results:**

Analyses were conducted on 67 participants in the intervention group (aged 56.7 ± 9.7 years; 71.6% female) and 60 participants in the active control group (aged 59.1 ± 8.4 years; 76.7% female). At 12 weeks, baseline adjusted mean change in systolic blood pressure (primary outcome) was greater in the active control group compared to the intervention group (-5.68 mmHg; 95% CI -10.86 to -0.50 mmHg; p = 0.03), but there were no differences between groups in mean change for secondary outcomes. Over 52-weeks, the difference in mean change for systolic blood pressure was no longer apparent between groups, but remained significant across the entire population (time: p < 0.001).

**Conclusions:**

In participants with increased cardiometabolic risk, exercise prescription alone had greater short-term improvements in systolic blood pressure compared to the mHealth intervention, though over 52 weeks, improvements were equal between interventions.

**Trial registration:**

ClinicalTrials.gov http://NCT01944124

**Electronic supplementary material:**

The online version of this article (doi:10.1186/1471-2458-14-1082) contains supplementary material, which is available to authorized users.

## Background

Cardiovascular diseases are the leading cause of morbidity and mortality world-wide [1] and patients with type 2 diabetes are at a high risk of developing cardiovascular complications. Metabolic syndrome is a clustering of risk factors that increases the risk of developing type 2 diabetes and cardiovascular diseases [2, 3]. Recent reports have shown that the prevalence of cardiovascular diseases, type 2 diabetes [4, 5] and metabolic syndrome [6] is higher in rural compared to urban settings. Much of the disparity is associated with demographic and behavioral factors associated with rural living [4, 6]. Evidence suggests that lifestyle interventions aimed at controlling cardiometabolic risk factors can reduce the incidence of type 2 diabetes [7, 8], but access to healthcare and lifestyle change programs is limited in rural communities. Novel interventions aimed at improving short-term uptake and long-term maintenance of such programs are needed for this population.

Mobile health (mHealth) has the potential to reach broad populations and may provide the opportunity to support lifestyle changes in rural populations. A number of studies have shown that mHealth is effective in the self-management of type 2 diabetes [9] and hypertension [10, 11]. Specifically, one study examining the effects of mHealth added to a hypertension management program showed improvements in both mHealth intervention and active control groups but greater improvements in BP in the mHealth intervention group [11]. These findings suggest that mHealth may have additional benefits when added to an existing treatment program; however, the use of mHealth to enhance exercise therapy has not been examined. Management of cardiometabolic risk factors prior to disease onset may have the potential to reduce disease burden. To date, however, evidence supporting the use of mHealth technologies for disease prevention is sparse. We recently completed a pilot study, which demonstrated that an intervention aimed at increasing physical activity while using mHealth technologies to monitor biometrics, improved diastolic blood pressure (DBP), total cholesterol and physical fitness over an eight-week period [12]. It remains unknown whether the mHealth tracking provided any added benefit over the exercise intervention alone and whether the improvements would be maintained over a longer follow-up period. The findings from this pilot study along with participant and clinician feedback was used to develop the intervention for the present study.

The purpose of this study was two-fold: first, to investigate the effects of a mHealth supported exercise intervention compared to an active control group receiving only the exercise prescription over a short-term (12-week) period; and second, to examine the long-term maintenance over 24 and 52 weeks of follow-up. The primary hypothesis was that the intervention group receiving mHealth support would have greater reductions in systolic blood pressure (SBP) and other cardiometabolic risk factors at 12 weeks compared to the active control group. Secondary hypotheses were that the mHealth intervention group would also have greater decreases in DBP, waist circumference, lipids (with the exception of high density lipoprotein cholesterol (HDL), which was expected to increase) and markers for blood glucose and inflammation compared to the active control group. It was hypothesized that all improvements would be better maintained over the 52-week follow-up period in the intervention group compared to the active control group.

## Methods

Full details of the study protocol have been previously published [13]. Community-dwelling adults aged 18-70 years from Huron-Perth and Grey-Bruce counties (Ontario, Canada) were recruited via print and radio advertisements, word of mouth, community presentations and physician referral. Participants with at least two metabolic syndrome risk factors as defined by the National Cholesterol Education Program Adult Treatment Panel III [waist circumference ≥88 cm (women) or 102 cm (men); SBP ≥135 mmHg and/or DBP ≥85 mmHg; fasting plasma glucose ≥6.1 mmol/L; fasting triglycerides ≥1.7 mmol/L; fasting HDL ≤1.29 mmol/L (women) or 1.02 mmol/L (men)] [14] presented to a community-based research centre (Gateway Rural Health Research Institute, Seaforth, Ontario, Canada) and voluntarily provided informed consent to participate in this parallel-group, randomized controlled trial (ClinicalTrials.gov NCT01944124). Exclusion criteria, evaluated by self-report during a screening phone call and verified in-person were: SBP >180 mmHg and/or DBP >110 mmHg; type 1 diabetes; history of myocardial infarction, angioplasty, coronary artery bypass or cerebrovascular ischemia; symptomatic congestive heart failure; atrial flutter; unstable angina; unstable pulmonary disease; use of medications known to affect heart rate; second or third degree heart block; history of alcoholism, drug abuse or other emotional cognitive or psychiatric problems; pacemaker; unstable metabolic disease; and orthopedic or rheumatologic problems that could impair the ability to exercise. This study was approved by the Western University Health Sciences Research Ethics Board (Protocol # 15828).

Participants (n = 149) were randomly assigned (1:1 allocation) to the mHealth supported exercise intervention group or the active control group. To accommodate deployment and activation of technology and implementation of technology training sessions, randomization was blocked based on appointment time, such that all participants attending appointments during week 1 were randomized to the intervention group, those attending appointments during week 2 were randomized to the control group, and so on. Due to this randomization procedure, research staff could not be blinded to group allocation. At baseline and at 12, 24, and 52 weeks of follow-up, participants reported to the clinic for assessment following a minimum eight-hour fast. Waist circumference was measured at the mid-point between the twelfth rib and upper border of the iliac crest following normal expiration. Supine blood pressure was measured with an automated sphygmomanometer (BPTru^®^, Coquitlam, Canada) following a five-minute rest period. Blood was drawn and sent to a central laboratory for analysis of fasting plasma glucose, triglycerides, cholesterol, glycated hemoglobin (HbA_1C_), insulin and high sensitivity C-reactive protein (CRP_hs_; a biomarker, which is increased with systemic inflammation). Insulin resistance was calculated using the Homeostatis Model Assessment for Insulin Resistance (HOMA-IR) [15].

Following a light, standardized snack, participants’ fitness was assessed and a tailored exercise program was prescribed by an exercise specialist according to the Step Test and Exercise Prescription (STEP™) protocol [16, 17]. Aerobic exercise was prescribed on most days of the week (5-7 days per week) at a training heart rate of 70, 75, 80 or 85% of an estimated maximum heart rate for those whose fitness was classified as poor, good, very good or excellent, respectively. Participants were to accumulate 30 to 60 minutes of exercise per day with bouts of at least 10 minutes each. Resistance training was prescribed two to four times per week. The exercise prescription was updated at 12 and 24 weeks. While the exercise specialist ensured that the exercise prescription complied with current global physical activity guidelines, which are recommended for optimal general health, including blood pressure control [18] (i.e., 150 minutes per week of moderate-to-vigorous intensity exercise), during the exercise counselling session, participants self-selected goals that were important to them. This was done in an attempt to motivate participants to adhere to the program to reach their own personal goals rather than attempting to encourage them with our pre-determined study goals. Participants were instructed to record all planned exercise sessions. Participants in the intervention group logged exercise using mHealth tools (described below) and the active control group logged exercise in a paper journal.

**Figure 1:**
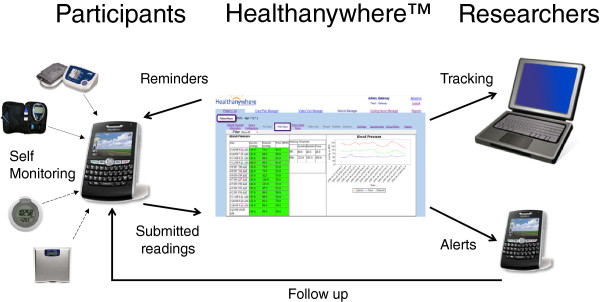
**Mobile health technologies.** Solid line = flow of information; dotted line = measurement transmitted from device to smartphone via Bluetooth^®^ connection; dashed line = measurement manually inputted to smartphone.

In addition to the exercise prescription, participants in the mHealth intervention group received a kit, which included a smartphone data portal (Blackberry^®^ Curve 8300 or 8530) equipped with Healthanywhere health monitoring application (Biosign Technologies Inc., Markham, Ontario, Canada), a Bluetooth™ enabled blood pressure monitor (A & D Medical, UA-767PBT, San Jose, California, USA), a glucometer (Lifescan One Touch Ultra2™, Milpitas, California, USA) with Bluetooth™ adapter (Polymap Wireless, PWR-08-03, Tucson, Arizona, USA) and a pedometer (Omron, HJ-150, Kyoto, Japan) (Figure 1). The mHealth technologies were provided primarily as a self-management tool. Participants attended a two-hour session, during which they learned how to use the technologies; were provided with information regarding normal values for fasting glucose, blood pressure and pedometer steps; and were encouraged to use the technologies to monitor their own health. Participants contacted research staff for troubleshooting as needed, but they did not receive additional lifestyle coaching compared to the active control group. The home-monitoring protocol required participants to input pedometer steps and exercise daily; measure blood pressure and fasting blood glucose three times per week; and measure body weight with their own home scale once per month. When measurements were outside of pre-determined safety limits, an automated alert was sent to the study physician’s smartphone for follow-up. Full details of database security, automated alerts and follow-up are reported elsewhere [13].

### Statistical analysis

The sample size was calculated based on an estimated difference in mean change between the intervention and active control groups in SBP (primary outcome measure) of 6 mmHg at 12 weeks. A common standard deviation of 12 mmHg was assumed. The sample size calculation assumed 80% power and two-sided significance level of 0.05. It was determined that 63 participants would be required per group and by assuming a 15% drop-out rate, the required sample size increased to 73 participants per group. Secondary outcomes were DBP, waist circumference, fasting plasma glucose, HbA_1C_, triglycerides, HDL, low density lipoprotein cholesterol (LDL), total cholesterol, HOMA-IR and CRP_hs_. An *a priori* decision was made to require a minimum of two data points (i.e., data from baseline visit and at least one follow-up visit) to impute missing data. All participants with complete data at baseline and at least one other time point (i.e., 12, 24 and/or 52 weeks) were included in the analyses (n =127). Last observation carried forward was used to impute missing data. For our primary analysis (12-week analysis for SBP), 2% of participants had their data carried forward from baseline. These participants had missed their 12-week visit but since they attended either the 24 and/or 52-week visit, data was carried forward from baseline (to 12-weeks) in these instances. For 12-week analysis of secondary outcomes, the proportion of participants with data carried forward ranged from 2 to 3%. For the longitudinal analyses, approximately 15% of participants had data carried forward in time.

Descriptive statistics were computed for baseline demographic and clinical characteristics at each time point by group. Specifically, means and standard deviations were calculated for continuous variables and frequency counts and percentages were calculated for categorical variables. Exercise compliance was calculated as the percentage of weeks in which at least 150 minutes of exercise was recorded. Compliance to the mHealth monitoring protocol was calculated as the percentage of measurements submitted for each monitoring tool over the 52-week intervention period and from weeks 1-12, 13-24 and 25-52. Analysis of covariance was used to examine differences between groups in mean change over 12 weeks, adjusting for baseline values. Thus, the outcome variable was change between baseline and 12-weeks and model terms included group and the baseline value of outcome. Two-way repeated measures ANOVA was used to extend the 12-week analyses to include data collected at all time-points, for each outcome of interest [i.e., four measurements in total, one corresponding to each time point, were included as part of the outcome variable]. For each model (outcome), the terms group, time, and group × time were included. Due to non-normality, triglycerides, HOMA-IR and CRP_hs_ were transformed to the natural logarithm scale for these analyses. Post-hoc comparisons were done using the Bonferroni method. Analyses were performed using R 3.0.1 [19].

## Results

### Participant flow

**Figure 2:**
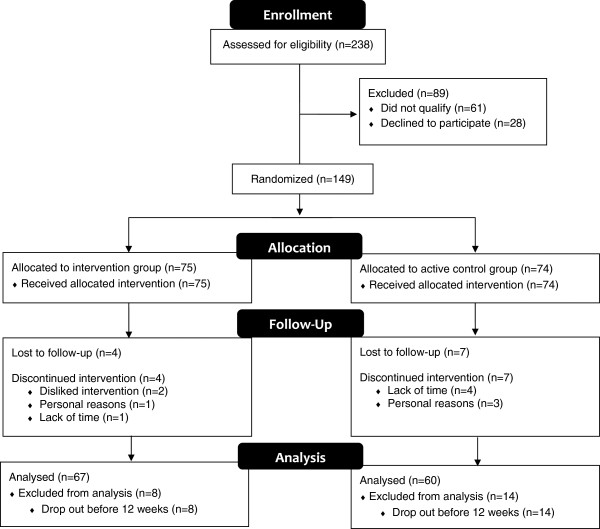
**Participant flow.**

During the recruitment period (November 2009 to December 2010) 238 individuals contacted the study coordinator to inquire about the trial and 89 individuals were excluded as they did not meet inclusion criteria (n = 61) or they were not interested in participating (n = 28). The remaining 149 individuals were randomized to either the technology intervention group (n = 75) or the active control group (n = 74). The flow of participants for the primary outcome analysis is shown in Figure 2. At 12 weeks, four participants in the intervention group and seven in the active control group failed to attend their appointment and were lost to follow-up. Four participants in the intervention group chose to discontinue the intervention because they disliked the technology intervention (n = 2), for personal reasons (n = 1) or due to lack of time (n = 1). Seven participants in the active control group chose to discontinue the intervention due to lack of time (n = 4) or personal reasons (n = 3). In total, eight participants were removed from analysis in the intervention group and fourteen from the active control group as only one data point was available (i.e., were lost to follow-up or withdrew from study after baseline assessment). Thus, 67 participants were analyzed from the intervention group and 60 participants were analyzed from the active control group.

Over the remainder of the follow-up period, three participants in the intervention group (n = 1 at 24 weeks; n = 2 at 52 weeks) and six in the active control group (n = 5 at 24 weeks; n = 1 at 52 weeks) failed to attend their appointment and were lost to follow-up. Three participants in the intervention group chose to discontinue the intervention due to lack of time (n = 1), personal (n = 1) or medical reasons (n = 1). Four participants in the active control group chose to discontinue the trial due to lack of time (n = 1), medical reasons (n = 2) or relocation to a different region (n = 1). The trial ended as per the protocol at the completion of the final 52-week follow up visit.

### Participant characteristics

Baseline demographic and clinical characteristics are shown in Table 1 for all participants who were randomized (n = 149). In both groups, participants were on average in their mid to late 50s, three-quarters were female and all were of Caucasian decent. At baseline, 27 (36.0%) and 33 (44.6%) of intervention and active control participants, respectively, reported antihypertensive therapy. Of these participants, 16 (59.3%) in the intervention group and 21 (63.6%) were prescribed only one antihypertensive medication, while the remaining were prescribed combination therapy. The median number of days per week of physical activity participation was 2.5 (IQR 3.5) for the intervention group and 3.0 (IQR 4.0) for the active control group. Average pVO_2max_ was 30.5 (6.8) ml/kg/min and 31.2 (6.3) ml/kg/min for the intervention and active control groups, respectively.

**Table 1:** Baseline Demographic and Clinical Characteristics*

Characteristic	Intervention (n =75)	Active Control (n =74)
Demographics		
Age (years)	55.7 (10.1)	57.8 (8.7)
Sex (female)	55 (73.3%)	56 (75.7%)
Race (Caucasian)^†^	74 (100%)	73 (100%)
Smoking status:		
Current	3 (4.0%)	1 (1.4%)
Former	31 (41.3%)	29 (39.2%)
History of Type 2 Diabetes	3 (4.0%)	4 (5.4%)
Antihypertensive Therapy	27 (36.0%)	33 (44.6%)
Monotherapy	16 (59.3%)	21 (63.6%)
Combination Therapy	11 (40.7%)	12 (36.3%)
Angiotensin Converting		
Enzyme Inhibitors	10 (37.0%)	20 (60.6%)
Angiotensin Receptor Blockers	6 (22.2%)	10 (30.3%)
Calcium Channel Blockers	4 (14.8%)	1 (3.0%)
Diuretics	17 (63.0%)	15 (45.4%)
Alpha Receptor Blockers	1 (3.7%)	0 (0%)
Average frequency of physical activity (in last 7 days), median (IQR)^‡^	2.5 (3.5)	3.0 (4.0)
pVO_2max_	30.5 (6.8)	31.2 (6.3)

*Abbreviations*: *IQR* interquartile range, *pVO*_*2max*_ predicted maximal oxygen uptake (aerobic fitness).

*Data are means (SD) or numbers (%) unless otherwise indicated. Percentages are calculated excluding missing values.

^†^Missing values: Intervention (n = 1) and Active Control (n = 1).

^‡^Average of: (a) number days (in last 7) that at least 30 minutes of continuous physical activity was done and b) number of days (in last 7) participated in a specific exercise session.

We compared these baseline demographic and clinical characteristics of the full study sample to those who remained in our main analyses (n =127; n =67 in the intervention group and n =60 in the active control group) and noted very similar percentages and descriptive statistics for both groups, for all characteristics.

### Short-term results

**Figure 3:**
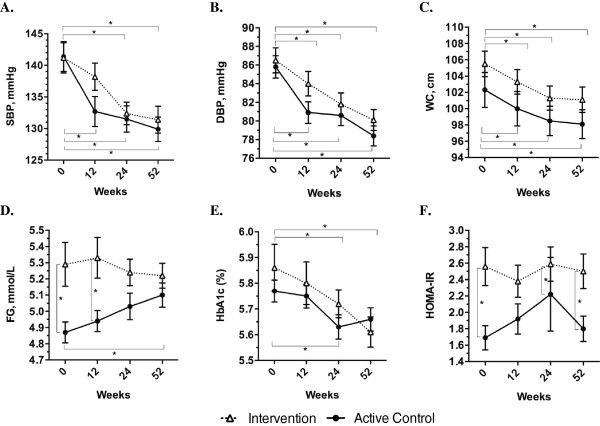
**Long-term results for blood pressure, waist circumference and glucose-related outcomes (Means ± SEM presented).** Presents change in **A)** systolic blood pressure (SBP); **B)** diastolic blood pressure (DBP); **C)** waist circumference (WC); **D)** fasting plasma glucose (FG); **E)** glycated hemoglobin (HbA1c); and **F)** homeostasis model for insulin resistance (HOMA-IR) over time. *p <0.05 (from post-hoc analyses using the Bonferroni method) White triangles = intervention group; black circles = active control group.

**Figure 4:**
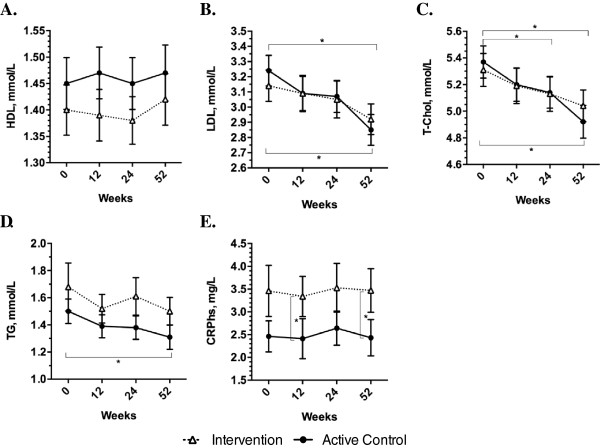
**Long-term results for lipid-related outcomes and CRP**_**hs**_ **(Means ± SEM presented).** Presents change in **A)** high density lipoprotein cholesterol (HDL); **B)** low density lipoprotein cholesterol (LDL); **C)** total cholesterol (T-Chol); **D)** triglycerides (TG); and **E)** high sensitivity C-reactive protein (CRPhs) over time. *p <0.05 (from post-hoc analyses using the Bonferroni method) White triangles = intervention group; black circles = active control group.

At 12 weeks, adjusted mean change in SBP was greater in the active control group compared to the intervention group (-5.68 mmHg; 95% CI -10.86 to -0.50 mmHg; p = 0.03) (Table 2). Post-hoc comparisons (following the two-way repeated measures ANOVAs) revealed within group differences from 0 to 12-weeks for the active control group only [Intervention: -2.96 (-7.48 to 1.57) mmHg, p = 0.35; Active Control: -8.73 (-13.6 to -3.91) mmHg, p < 0.001]. For all secondary outcomes examined, there were no group differences in 12-week mean change (Table 2). Although there were no other differences between groups at 12-weeks, post-hoc comparisons revealed within group differences for both groups from 0 to 12-weeks for DBP [Intervention: -2.58 (-4.90 to -0.27) mmHg, p = 0.02; Active Control: -4.86 (-7.33 to -2.40) mmHg, p < 0.001], and waist circumference [Intervention: -0.87 (-1.63 to -0.11) inches, p = 0.02; Active Control: -0.91 (-1.71 to -0.11) inches, p = 0.02]. Graphical results are displayed in Figures 3 and 4 for the primary outcome and all secondary outcomes.

**Table 2:** **Short-term Results: Primary and Secondary Outcomes**

	Intervention (n = 67)		Active Control (n = 60)		Difference in Mean Change at 12-weeks ^*^	
Outcome measure	Baseline Mean (SD)	12-weeks Mean (SD)	Baseline Mean (SD)	12-weeks Mean (SD)	Estimate (95% CI)	***p***
SBP_rest_, mmHg^†^	141.2 (19.7)	138.2 (17.8)	141.4 (18.2)	132.7 (18.4)	-5.68 (-10.86 to -0.50)	0.03
DBP_rest_, mmHg^†^	86.5 (11.0)	84.0 (10.7)	85.8 (9.3)	80.9 (9.0)	-2.55 (-5.24 to 0.13)	0.06
WC, cm	105.5 (12.8)	103.3 (12.3)	102.3 (16.6)	100.0 (16.4)	-0.36 (-2.18 to 1.46)	0.69
FG, mmol/L	5.29 (1.11)	5.33 (1.03)	4.87 (0.50)	4.94 (0.51)	-0.06 (-0.24 to 0.11)	0.47
HbA1c, %	5.86 (0.75)	5.80 (0.68)	5.77 (0.33)	5.75 (0.36)	0.02 (-0.07 to 0.10)	0.65
HbA1c, (mmol/mol)	41 (8.2)	40 (7.4)	40 (3.6)	39 (3.9)	0.2 (-0.8 to 1.1)	0.65
HOMA-IR^‡^	2.56 (1.88)	2.38 (1.59)	1.69 (1.15)	1.92 (1.44)	0.12 (-0.30 to 0.53)	0.58
HDL, mmol/L	1.40 (0.39)	1.39 (0.40)	1.45 (0.38)	1.47 (0.38)	0.04 (-0.03 to 0.10)	0.26
LDL, mmol/L	3.14 (0.84)	3.09 (0.96)	3.24 (0.78)	3.09 (0.87)	-0.08 (-0.31 to 0.15)	0.51
T-Chol, mmol/L	5.31 (1.01)	5.19 (1.09)	5.37 (0.94)	5.20 (0.98)	-0.05 (-0.30 to 0.21)	0.72
TG, mmol/L	1.68 (1.45)	1.52 (0.87)	1.50 (0.70)	1.39 (0.66)	-0.04 (-0.20 to 0.13)	0.67
CRP_hs_, mg/L	3.46 (4.59)	3.34 (3.61)	2.46 (2.64)	2.41 (3.42)	-0.16 (-0.84 to 0.53)	0.65

*Abbreviations*: *CI* Confidence interval, *SBP*_*rest*_ resting systolic blood pressure, *DBP*_*rest*_ resting diastolic blood pressure, *WC* waist circumference, *FG* fasting glucose, *HbA*_*1c*_ glycated hemoglobin, *HOMA-IR* Homeostasis Model for Insulin Resistance, *HDL* high density lipoprotein cholesterol, *LDL* low density lipoprotein cholesterol, *T-Chol* total cholesterol, *TG* triglycerides, *CRP*_*hs*_ high sensitivity C-reactive protein.

^*^Group differences calculated as Active Control – Intervention and adjusted for baseline.

^†^n =59 in the active control group.

^‡^n =65 in the intervention group.

### Long-term (Maintenance) results

When all time-points were considered, the difference in mean change for SBP was no longer apparent between the intervention and active control groups, however, the decrease remained significant across the entire population (*time*: p < 0.001). Post-hoc comparisons revealed that there were significant differences from 0 to 24 and 52 weeks within both groups (see Figure 3 Part A, all p < 0.001). For the secondary outcomes over the 52-week follow-up period, there was a main effect of *time* for DBP, waist circumference, HbA_1C_, LDL, total cholesterol, and triglycerides but there were no *group* or *group × time* effects. There was a main effect for *group* for fasting plasma glucose, HOMA-IR and CRP_hs_, which were all higher in the intervention group, and there was a *group × time* interaction for fasting plasma glucose. There were no significant effects for HDL (see Additional file 1: Table S1).

Results of post-hoc analyses for within group changes over time and between group changes at each time point are shown on the plots in Figures 3 and 4. DBP and waist circumference were reduced in both intervention and active control groups at 24 and 52 weeks compared to baseline (all p < 0.001). There were no changes in fasting plasma glucose in the intervention group, but compared to baseline, fasting plasma glucose was increased at 52 weeks in the active control group (p = 0.02). At baseline and 12-weeks, fasting plasma glucose was higher in the intervention compared to the active control group (both p < 0.05), but there were no differences between groups at 24 or 52 weeks (see Figure 3 Part D). Compared to baseline, HbA_1C_ was reduced in both intervention and active control groups at 24 weeks (p < 0.05), but at 52 weeks it remained lower than baseline only in the intervention group (p < 0.001) (see Figure 3 Part E). At baseline, 24 and 52 weeks, HOMA-IR was higher in the intervention group compared to the active control group (all p < 0.05), with no differences at 12 weeks (see Figure 3 Part F). At 52 weeks, LDL was reduced in both intervention and active control groups compared to baseline (p < 0.05), and total cholesterol was reduced in the active control group at 24 weeks (p = 0.04) and in both groups at 52 weeks (p < 0.01), compared to baseline (see Figure 4 Parts B and C). Triglycerides were reduced only in the active control group at 52 weeks compared to baseline (p = 0.005) (see Figure 4 Part D). Finally, CRP_hs_ was higher in the intervention group at 12 weeks (p = 0.02) and 52 weeks (p = 0.02), with no differences at baseline and 24 weeks (see Figure 4 Part E).

### Compliance to exercise prescription

Exercise compliance is presented as mean (standard deviation; SD). At 12 weeks, the intervention and active control groups exercised on average 188.2 (189.5) and 170.3 (161.2) minutes per week, respectively. Exercise compliance was 47.8% (41.2%) for the intervention group and 46.1% (39.0%) for the active control group. Twenty-seven (40%) participants in the intervention group and 18 (30%) of participants in the active control group had an exercise compliance of 75% or greater.

### Compliance to mHealth Self-management protocol

Compliance is presented as mean (SD). Over the 52-week intervention period 82.7 (21.8), 82.2 (22.2), 70.9 (28.4) and 41.5 (29.8)% of measurements were completed for BP, FPG, pedometer steps and body weight, respectively. BP monitoring compliance decreased from 91.5 (17.4)% from weeks 1-12 to 86.7 (20.3)% from weeks 13-24 and to 77.6 (27.5)% from weeks 25-52. Similarly, compliance to the FPG monitoring protocol decreased from 90.3 (17.8)% from weeks 1-12 to 87.2 (20.0)% from weeks 13-24 and to 77.0 (27.9)% from weeks 25-52. Compliance to the activity monitoring protocol by logging pedometer steps decreased from 83.6 (21.1)% from week 1-12 to 77.2 (28.4)% from weeks 13-24 to 63.1 (34.9)% from weeks 25-52. Body weight measurement compliance decreased from 63.6 (31.9)% from weeks 1-12 to 38.2 (40.4)% from weeks 13-24 and to 28.4 (34.2)% from weeks 25-52.

### Alarms

Over the 52-week intervention period, 12 alarms were triggered from high glucose readings, with the majority (n = 11) from a single participant who was diagnosed with type 2 diabetes after following up with her family physician in response to the high readings in the study. Seven alarms were triggered from high diastolic BP readings, one which resulted in physician follow-up and increased medication. One alarm was triggered from a mistakenly high heart rate reading, but none from systolic BP.

### Adverse events

Four adverse events were reported from three participants in the active control group. Adverse events included angina (n = 2), stroke (n = 1), and arm/shoulder pain (n = 1). No adverse events were reported in the intervention group.

## Discussion

Contrary to the hypothesis, the main finding of this study was that at 12 weeks, the reduction in SBP was greater in the active control group compared to the intervention group. By 52 weeks, however, the reduction in SBP compared to baseline was similar between groups. At 12 weeks, the mean change in all secondary outcomes examined was similar between groups. Over the 52-week follow up period, the improvements in DBP, waist circumference, HbA_1C_, LDL and total cholesterol were similar between groups, with no change in HDL for either group.

This study was limited to highly motivated adults residing in rural communities who volunteered to participate in an intensive lifestyle intervention. Therefore, findings may not be generalizable to less motivated adults or to those residing in urban centres. Since the study was conducted in small rural communities and a considerable amount of recruitment was accomplished by word-of-mouth, it is likely that there was contact between participants in the mHealth intervention and active control groups throughout the course of the study. Social support was not controlled for and may have biased results. The randomization procedures were pragmatically designed for deployment of technology kits and activation of smartphones and the mobile application. While a more typical randomization procedure would have made the study design stronger (i.e., completing randomization after baseline assessments), it would have been impractical due to the logistics associated with implementing the technology component. Although the primary outcome measure was SBP, blood pressure reduction was not necessarily the goal of the exercise program. Rather, participants’ self-selected goals and the exercise specialist provided guidance and prescribed an exercise program to achieve these goals; had all participants received exercise prescriptions based on the study goals, greater improvements may have occurred. Tailored exercise programs, however, are generally more effective and participants may have been less motivated to adhere to their exercise prescription if it had not been based on their personal health goals. In fact, the STEP™ tool has been investigated in previous randomized clinical and observational studies and has been proved to positively effect exercise behaviours and cardiometabolic risk factors [17]. The STEP™ protocol requires little equipment and can be easily translated to community settings. The mHealth monitoring technology and protocol was tested for feasibility and the development of this protocol was guided by our previous pilot study [12, 20].

Lifestyle modification is known to improve cardiometabolic risk status and is recommended as first line therapy for prevention and treatment of metabolic syndrome [2, 3, 14]. Longitudinal studies have demonstrated the efficacy of lifestyle modification interventions targeting physical activity, diet and other behaviours to improve metabolic syndrome risk factors [21]. Such programs, however, are often not available to rural populations. mHealth-supported interventions are accessible to a broad population, including those in rural communities, and have the potential to be delivered as sustainable programs for behaviour modification and maintenance. Research supports the use of electronic health, including mHealth interventions for management of diabetes [9] and hypertension [10, 11]. Contrary to these findings, and those of a meta-analysis that examined the effects of long-term exercise interventions on metabolic syndrome and showed reductions in fasting plasma glucose [22] the present study did not find positive effects on fasting plasma glucose. HbA_1C_, which is a marker of three-month glucose regulation, showed a different trend with small, but significant reductions in the mHealth intervention group at 24 and 52 weeks and a transient reduction in the active control group at 24 weeks. Average values for both groups over the 52-week period, however, remained within the clinically defined healthy range, which may have limited the potential for improvement. Both SBP and DBP, on the other hand, were improved over the course of the 52-week study, though the active control group had greater improvements in SBP at 12 weeks compared to the intervention group. This result is supported by a recent meta-analysis that showed that internet-based blood pressure control interventions were more effective when they were at least six months in duration compared to those of shorter duration [10]. Similar analyses have not been completed for smartphone interventions, but a longer time period may be required to elicit change. Since the active control group received only the tailored exercise prescription, these participants may have been more focused on completing exercise sessions, while the intervention group may have been initially overwhelmed with the exercise prescription and mHealth technology. Once their comfort with the mHealth monitoring protocol increased, they may have changed their focus to the exercise prescription. Exercise compliance over the first 12 weeks was, however, similar between groups. Contrary to the hypothesis and results of similar interventions [11] there were no between group differences in SBP or DBP at 24 or 52 weeks. In another year-long intervention study for patients with diabetes and uncontrolled hypertension, the intervention group, which received immediate feedback regarding submitted blood pressure measures, reduced daytime ambulatory SBP by 9 mmHg, while the control group who monitored blood pressure but did not receive feedback, did not experience improvements [11]. Targeted feedback may be an important component to mHealth interventions. Although participants in our study were able to review past measurements and trends on their own, tailored feedback messages might have resulted in greater improvements in metabolic risk factors in the mHealth intervention group compared to the active control group.

mHealth has been suggested as an alternate or even replacement for traditional clinical management of chronic disease risk factors [23, 24]. Access and availability of care could include mHealth solutions among patients and health care providers. Such considerations currently lack rigorous investigation. In this study, both the mHealth intervention and active control groups showed improvements in cardiometabolic risk factors over the course of the study, highlighting the importance of exercise prescription for cardiometabolic risk management. An interesting finding in the present study was that, contrary to the hypothesis, SBP was initially reduced (at 12-weeks) to a greater extent in the active control group compared to the mHealth technology intervention group, though there was no difference between groups at 24 and 52 weeks of follow-up. While more research is needed, it may be that an exercise program alone should be prescribed at the onset of risk management and mHealth could be added as a tool to aid in long-term maintenance of behavior change. As discussed above, more sophisticated applications involving immediate automated feedback may be more effective for supporting optimal health behaviours. Additionally, with the exponentially increasing number of health management applications available for smartphones, clinicians and patients could chose those that specifically suit management and lifestyle needs and preferences.

Although the differences in change in cardiometabolic risk status were similar between the mHealth intervention and active control groups, this study provides insight to guide design and implementation of future, more sophisticated mHealth applications, which may lead to improved long-term management of chronic disease risk factors. The ideal protocol for mHealth monitoring remains unknown, though it is likely that it should be tailored to suit individuals’ lifestyle preferences and risk factor modification needs. Additionally, it is unclear why the active control group reduced SBP at 12 weeks while the mHealth intervention group did not realize improvements until 24 weeks. While these findings correspond with those from internet-based health intervention studies, research is needed to examine the underlying reasons for the extended timeline for SBP reductions to enable implementation of interventions for optimal risk management.

## Conclusions

In conclusion, there appears to be no clear advantage of a mHealth-supported exercise intervention in patients with increased cardiovascular risk, over an active control group receiving the STEP™ exercise prescription alone. In fact, the STEP™ exercise prescription alone elicited greater reductions in SBP after 12 weeks compared to the mHealth intervention group. Over the long-term, however, changes in SBP and other cardiometabolic risk factors were equal between groups, suggesting that short-term improvements in cardiometabolic risk may be more effective with simpler interventions, but long-term improvements can be realized with both exercise prescription and mHealth supported exercise prescription. Perhaps an exercise prescription following the STEP™ protocol is best to achieve the initial SBP reduction and then mHealth tracking could be offered as an assistive tool for interested patients or for patients in need of monitoring for control of other diseases such as hypertension or pre-diabetes.

## Electronic supplementary material

Additional file 1: **Longitudinal Results for Clinical and Physiological Outcomes: Overall Findings** ^**a**^ **.** (DOCX 14 KB)

## Abbreviations

CRP_hs_: High sensitivity C-reactive protein
DBP: Diastolic blood pressure
HbA_1C_: Glycated hemoglobin
HDL: High density lipoprotein cholesterol
HOMA-IR: Homeostasis model of assessment, insulin resistance
LDL: Low density lipoprotein cholesterol
mHealth: Mobile health
SBP: Systolic blood pressure
STEP™: Step test and exercise prescription.
