# Supplementary material for: Mobile health, exercise and metabolic risk: a randomized controlled trial
Source: BMC Public Health. 2014 Oct 18;14:1082. doi: 10.1186/1471-2458-14-1082 (PMC4210561; doi:10.1186/1471-2458-14-1082)
Supplement: Supplementary file 1 — Additional file 1: Longitudinal Results for Clinical and Physiological Outcomes: Overall Findings a . (DOCX 14 KB) [file 12889_2014_7183_MOESM1_ESM.docx]

**Supplement 1. Longitudinal Results for Clinical and Physiological Outcomes: Overall Findings^a^**

|  |  | **Factors** | | | | | |
| --- | --- | --- | --- | --- | --- | --- | --- |
|  |  | **Group** | | **Time** | | **Group × Time** | |
| **Outcomes** | **N** | **F value** | **p** | **F value** | **p** | **F value** | **p** |
| SBP_rest,_ mmHg | 126 | 0.56 | 0.46 | **23.92** | **<0.001** | 1.68 | 0.17 |
| DBP_rest_, mmHg | 126 | 1.28 | 0.26 | **34.5** | **<0.001** | 0.97 | 0.41 |
| WC, cm | 127 | 1.65 | 0.20 | **23.46** | **<0.001** | 0.07 | 0.98 |
| FG, mmol/L | 127 | **6.34** | **0.01** | 0.63 | 0.60 | **3.11** | **0.03** |
| HbA1c, % | 127 | 0.33 | 0.57 | **12.7** | **<0.001** | 1.85 | 0.14 |
| HOMA-IR ^b^ | 125 | **9.48** | **0.003** | 0.71 | 0.55 | 0.67 | 0.57 |
| HDL,mmol/L | 127 | 0.98 | 0.33 | 1.37 | 0.25 | 0.66 | 0.58 |
| LDL, mmol/L | 125 | 0.01 | 0.93 | **10.10** | **<0.001** | 0.69 | 0.56 |
| T-Chol, mmol/L | 127 | 0.002 | 0.97 | **10.35** | **<0.001** | 0.74 | 0.53 |
| TG, mmol/L^b^ | 127 | 1.16 | 0.28 | **3.09** | **0.03** | 1.07 | 0.36 |
| CRP_hs_, mg/L^b^ | 127 | **6.68** | **0.01** | 0.49 | 0.69 | 1.38 | 0.25 |

**Abbreviations:** SBP_rest_ = resting systolic blood pressure; DBP_rest_= resting diastolic blood pressure; WC = waist circumference; FG = fasting glucose; HgA_1c_ = glycated hemoglobin; HDL = high density lipoprotein cholesterol, LDL = low density lipoprotein cholesterol, T-Chol = total cholesterol; TG = triglycerides; HOMA-IR = Homeostasis Model for Insulin Resistance; CRP_hs_ = high sensitivity C-reactive protein.

^a^ Two-way Analysis of Variance (ANOVA) with One Repeated Factor performed for each outcome

^b^ Outcome transformed to the natural logarithm scale due to non-normality on original scale
